# Enhancing cancer‐associated fibroblast fatty acid catabolism within a metabolically challenging tumor microenvironment drives colon cancer peritoneal metastasis

**DOI:** 10.1002/1878-0261.12917

**Published:** 2021-02-16

**Authors:** Shaoyong Peng, Daici Chen, Jian Cai, Zixu Yuan, Binjie Huang, Yichen Li, Huaiming Wang, Qianxin Luo, Yingyi Kuang, Wenfeng Liang, Zhihang Liu, Qian Wang, Yanmei Cui, Hui Wang, Xiaoxia Liu

**Affiliations:** ^1^ Department of Colon and Rectum Surgery The Sixth Affiliated Hospital (Guangdong Gastrointestinal and Anal Hospital) Sun Yat‐sen University Guangzhou China; ^2^ Guangdong Provincial Key Laboratory of Colorectal and Pelvic Floor Disease The Sixth Affiliated Hospital (Guangdong Gastrointestinal and Anal Hospital) Sun Yat‐sen University Guangzhou China; ^3^ Department of Clinical Laboratory The Sixth Affiliated Hospital Sun Yat‐sen University Guangzhou China

**Keywords:** CAF, colorectal cancer, CPT1A, FAO, glycolysis, peritoneal metastases

## Abstract

Most cancer‐related deaths result from the progressive growth of metastases. Patients with peritoneal metastatic (PM) colorectal cancer have reduced overall survival. Currently, it is still unclear why colorectal cancer (CRC) cells home to and proliferate inside the peritoneal cavity, and there is no effective consolidation therapy for improved survival. Using a proteomic approach, we found that key enzymes of fatty acid oxidation (FAO) were decreased in patients with PM colorectal cancer. Furthermore, we confirmed that carnitine palmitoyltransferase IA (CPT1A), a rate‐limiting enzyme of FAO, was expressed at significantly low levels in patients with PM colorectal cancer, as determined by RT‐qPCR, IHC, and GEO dataset analysis. However, lipidomics revealed no difference in FFA levels between PM and non‐PM primary tumors. Here, we showed that cancer‐associated fibroblasts (CAFs) promote the proliferation, migration, and invasion of colon cancer cells via upregulating CPT1A to actively oxidize FAs and conduct minimal glycolysis. In addition, coculture‐induced glycolysis increased in cancer cells while fatty acid catabolism decreased with lower adiponectin levels. Importantly, inhibition of glycolysis significantly reduced the survival of CRC cells after incubation with conditioned medium from CAFs*^CPT1A^* ^‐OE^ *in vitro* and impaired the survival and growth of organoids derived from CRC‐PM. Finally, we found that directly blocking FAO in CAFs*^CPT1A^* ^‐OE^ with etomoxir inhibits migration and invasion *in vitro* and decreases tumor growth and intraperitoneal dissemination *in vivo*, revealing a role for CAF CPT1A in promoting tumor growth and invasion. In conclusion, our results suggest the possibility of testing FAO inhibition as a novel approach and clinical strategy against CAF‐induced colorectal cancer with peritoneal dissemination/metastases.

## Graphical abstract

Patients with peritoneal metastatic (PM) colorectal cancer have reduced overall survival. In metabolically challenging tumor microenvironment, cancer‐associated fibroblasts (CAFs) enhance fatty acid catabolism by upregulating CPT1A expression and drive colon cancer peritoneal metastasis. Our results suggest the possibility of testing fatty acid oxidation inhibition as a novel approach and clinical strategy against CAF‐induced colorectal cancer with peritoneal dissemination/metastases.

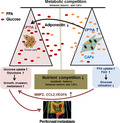

**Abbreviations**
3‐BrPA: 3‐bromopyruvate
CAFs: cancer‐associated fibroblasts
CM: conditioned medium
CPT1A: carnitine palmitoyltransferase I A
CRC: colorectal cancer
CRS: cytoreductive surgery
ECAR: extracellular acidification rate
ETO: etomoxir
FAO: fatty acid oxidation
FFA: free fatty acid
GEO: gene expression omnibus
HIPEC: hyperthermic intraperitoneal chemotherapy
IHC: immunohistochemistry
KD: knockdown
OA: oleic acid
OCR: oxygen consumption rate
OE: overexpression
PM: peritoneal metastatic
TCGA: The Cancer Genome Atlas
TME: tumor microenvironment
VC: vector control
α‐SMA: α‐smooth muscle actin

## 1 Introduction

Worldwide, colorectal cancer (CRC) is the third most commonly diagnosed malignancy and the second leading cause of cancer‐related death [1]. CRC metastasis is a major contributor to cancer‐related mortality. Patients with peritoneal metastatic (PM) colorectal cancer have a poorer prognosis and significantly shorter overall survival than patients with other types of CRC [2, 3]. Currently, it is still unclear why CRC cells home to and proliferate in the peritoneum. However, cytoreductive surgery (CRS) and hyperthermic intraperitoneal chemotherapy (HIPEC) may prolong survival time and increase quality of life for patients with peritoneal metastasis from colorectal cancer (PM‐CRC), but not clinical routine everywhere (e.g., small volume hospitals).

The altered lipid metabolism in cancer has gained increasing attention because lipids are major components of biological membranes, are used in cellular metabolism and energy storage, and serve key roles as signaling molecules [4, 5]. Indeed, fatty acid metabolisms contribute to tumor cell growth and survival [6, 7, 8]. Sounni et al demonstrated tumor adaptation to VEGF blockade through a metabolic shift toward carbohydrate and lipid metabolism and further showed that blocking lipid synthesis can effectively inhibit tumor regrowth and metastasis after antiangiogenic therapy withdrawal [9]. Iwamoto et al showed that lipid‐dependent metabolic reprogramming in tumor cells confers antiangiogenic drug (AAD) resistance by switching from glycolysis to FAO metabolism upon AAD treatment, which can trigger hypoxia [10]. In addition, Nieman et al indicated that adipocytes promote the initial homing of tumor cells to the omentum by providing fatty acids to the cancer cells, fueling rapid tumor growth [6]. However, the underlying mechanism of the lipid metabolism and peritoneal metastasis of CRC remains incompletely understood. We hypothesized that altered lipid metabolism in the microenvironment may play an essential role in CRC with peritoneal metastasis.

## 2 Materials and methods

### 2.1 Patient sample collection and cultivation of primary human fibroblasts

All primary tumors were collected via the resection from chemo‐naïve patients with T4Nx colorectal cancer (age 18–70), which were obtained from the Sixth Affiliated Hospital of Sun Yat‐sen University (SYSU, China). Informed consent was obtained before surgery, and the study was approved by the IRB of the Sixth Affiliated Hospital of Sun Yat‐sen University. Primary human carcinoma‐associated fibroblasts (CAFs) were isolated from tumor samples as previously described [11]. Isolated CAFs were cultured in DMEM with 10% FBS and 1% penicillin/streptomycin. To generate conditioned media, CAFs were seeded into 10 cm dishes and allowed to reach 90% confluence or treated with ETO/OA for 24 h. The medium was replaced with complete DMEM supplemented with 1% pen/strep, and the cells were incubated for 24–36 h. Media were collected, filtered with a 0.8‐mm filter to remove cell debris, and then used. Control CM was generated by incubating complete DMEM supplemented with 1% pen/strep for the same time in a dish without cells. HCT116 and DLD1 cells (ATCC) were cultured in McCoy's 5A and RPMI‐1640 (GIBCO, Life Technologies, NY, USA) with 10% fetal bovine serum (FBS) (GIBCO, Life Technologies, NY, USA), respectively. Cell lines were cultured in an incubator at 37 °C with 5% CO_2_. All cell lines were regularly tested for mycoplasma (every 3–4 months). The study methodologies conformed to the standards set by the Declaration of Helsinki.

### 2.2 Database analysis

The Gene Expression Omnibus (GEO) database and The Cancer Genome Atlas (TCGA) database were used to analyze the potential correlation between CPT1A expression in CRC patients and their survival. The hazard ratio was estimated by fitting a Cox proportional hazard model (Surv(time, status)) using the risk group as the covariate.

### 2.3 Metabolomics

*CPT1A* ^VC^/*CPT1A* ^KD^ DLD1 cells were collected, and then, metabolites were extracted. Briefly, cells (5*10^6^) were pelleted (5 min at 14 000 ***g***, 4 °C), washed with cold PBS, and snap‐frozen in liquid nitrogen. Quintuplicate samples were collected and sent for analysis by Metabolon‐associated energy metabolism (Applied Protein Technology, Shanghai, China), as described in a previous publication [12].

### 2.4 Lipidomics methods

Sample was prepared, and lipids were extracted according to the methyl tert‐butyl ether (MTBE) method. Briefly, samples were homogenized with 200 µL water and 240 µL methanol (Thermo Fisher, Waltham, MA, USA). Then, 800 µL of MTBE was added and the mixture was ultrasound for 20 min at 4 ℃ followed by sitting still for 30 min at room temperature. The solution was centrifuged at 14 000 ***g*** for 15 min at 10 ℃, and the upper organic solvent layer was obtained and dried under nitrogen, and stored at −80 ℃ until use. The lipid analysis was performed with LC‐MS/MS, as described in a previous publication [13]. The lipidomics was done by Applied Protein Technology (Shanghai, China).

### 2.5 Multilabeling and confocal microscopy

For immunofluorescence double labeling, tumors of frozen colorectum sections were costained with CPT1A (Abcam, Cambridge, MA, USA) and α‐SMA (Cell Signaling Technologies, Danvers, MA, USA) antibodies. Then, sections were incubated with a primary antibody at 4 °C overnight and rinsed in PBS, followed by incubation with the appropriate Alexa Fluor 488‐ and Alexa Fluor 546‐conjugated secondary antibodies. Sections were washed in PBS, mounted in aqueous mounting media with DAPI, and visualized and imaged under a confocal microscope (ZEISS LSM 880).

### 2.6 Immunohistochemistry

Tumor tissues were fixed with 10% formaldehyde and embedded in paraffin. In brief, paraffin‐embedded specimens were cut into 4‐mm sections, deparaffinized, antigen‐retrieved by microwave oven heating, and then incubated overnight with monoclonal antibodies against human CPT1A (1 : 1000) (Abcam) or α‐SMA (1 : 200) (Cell Signaling Technologies). Biotinylated secondary antibody was then added and incubated for 1 h at 37 °C. After washing with PBS, the slides were incubated with 3, 3‐diaminobenzidine tetrahydrochloride (DAB) substrate, washed, and examined under a light microscope, as described previously [14].

### 2.7 Forced overexpression of CPT1A

CPT1A plasmid and the empty vector control were obtained from GeneCopoeia (Guangzhou, China). Recombinant plasmids together with lentivirus packaging plasmids were cotransfected into 293T cells to obtain lentivirus particles, and the virus titer was determined. CAF cells were seeded in 60‐mm dishes per replicate at a density of 1 × 10^6^ cells·mL^−1^ in 10 mL of complete medium per dish and infected with lentivirus particles at a multiplicity of infection (MOI) of 0.3 in the presence of polybrene (4 µg·mL^−1^). Stable CPT1A‐overexpressing CAF cells were selected in cell culture medium containing 1.5 μg·mL^−1^ puromycin (Selleck, Houston, TX, USA), as described previously [14]. The protein levels of CPT1A were detected by western blot.

### 2.8 Knockdown assays

Lentiviral particles including shRNAs against CPT1A were obtained from GeneCopoeia: target sequence 1: GCTCTTAGACAAATCTATCTC, and target sequence 2: GCCTTTGGTAAAGGAATCATC.

### 2.9 Measurement of intracellular ATP levels

The level of intracellular ATP was measured by the CellTiter‐Glo Luminescent Cell Viability Assay (Promega, Madison, WI, USA), as previously described [15]. The luminescence was detected by Thermo Scientific Varioskan Flash Multiplate Reader (Thermo Fisher Scientific).

### 2.10 Real‐time bioenergetics analysis

Oxygen consumption rates (OCRs) and extracellular acidification rates (ECARs) were measured using an XF24 extracellular analyzer (Seahorse Bioscience, USA), as described previously [16].

### 2.11 Glucose uptake and lactate production

The glucose and lactate levels were determined by using an SBA‐40C Biosensor (Biology Institute of the Shandong Academy of Science, Jinan, Shandong Province, China), as described previously [17].

### 2.12 Cell migration and invasion assays

Transwell assays were performed with 24‐well cell culture inserts with an 8.0 µm pore size (Falcon, USA) with Matrigel (1 : 10) (BD Biosciences, USA). A total of 5 × 10^4^ DLD1 or 8 × 10^4^ HCT116 cells were placed into the upper chamber in 0.2 ml of serum‐free DMEM, whereas CAF cells or CAF CM or control medium was placed in the lower chamber as a chemoattractant. Migrated and invaded cells on the other side of the membrane were fixed and stained with crystal violet for 5 min after 24 h of culture. Each assay was repeated three times. For the wound healing assay, 5 × 10^4^ cells per well in 4‐well culture inserts (ibidi, Germany) were seeded into 12‐well plates, and wounds were made. The size of the wound was captured every day by an IncuCyte ZOOM live cell imager and measured by ImageJ software.

### 2.13 Cell proliferation assay: CCK‐8 and IncuCyte ZOOM

Cell proliferation was assessed with Cell Counting Kit‐8 (CCK‐8) (Dojindo Lab, Japan) according to the manufacturer’s instructions. In brief, a total of 5000 cells/well were seeded into 96‐well plates, and then, cells were cultured in the indicated conditions for 72h, and the proliferation index at 450 nm absorbance was measured by a Thermo Scientific Varioskan Flash instrument after incubation with 10 µl CCK‐8 solution for 2 h at 37 °C. Cell proliferation was also assessed via IncuCyte ZOOM (Essen BioScience). A total of 5000 cells/well were seeded into 96‐well plates and were automatically monitored and recorded every 2 h by the IncuCyte ZOOM for 72h.

### 2.14 Organoid culture

Tissue samples from PMs were collected during cytoreductive surgery (CRS). Five tissue samples of colorectal peritoneal metastases were collected for organoid derivation. From these samples, 3 different organoid lines were established (Accuroid™, Accurate Biotechnology, China). For drug screening purposes, organoids were dispensed through enzymatic and mechanical force. Cell‐Matrigel suspensions were placed into 96‐well plates, and treatment was initiated 24 hours after plating. After 3 days of treatment, the organoids were imaged.

### 2.15 Mouse models

For the subcutaneous model, 3.6 × 10^5^ primary CAFs expressing a control vector (n = 4) or a CPT1A overexpression vector were mixed with 1.8 × 10^5^ DLD1 cells and then injected subcutaneously into the flanks of 6‐week‐old female athymic nude mice (n = 4). DLD1 cells were injected alone as a control. The tumors were measured every 2–3 days using calipers, and the tumor volumes were calculated using the following standard formula: width^2^ × length × 0.52.

For the *in vivo* metastasis assay with conditioned media, 1 × 10^6^ HCT116‐Luc + cells were injected intraperitoneally into 6‐week‐old athymic nude female mice in 200 µl of conditioned media from 4 different conditions as described in Fig. 7A. One week and 2 weeks later, mice were imaged using an IVIS Spectrum Imaging System (Caliper Life Sciences, Hopkinton, MA), respectively.

### 2.16 Luminex‐based assays: multiple soluble molecule analysis

The concentration of soluble molecules in suspension medium or tissue lysates was measured using Human Premixed Multi‐Analyte Kits (R&D Systems, Minneapolis, MN, USA). Human Premixed Multi‐Analyte Kit 1 assesses 9 cytokines: CCL2, EGF, MIF, TNF‐a, IFN‐γ, IL‐2, IL‐10, IL‐12/23 p40, VEGF‐A; Human Premixed Multi‐Analyte Kit 2 assesses 3 cytokines: adiponectin/Acrp30, MMP2, and MMP9. Samples were assayed according to the manufacturer’s instructions. Fluorescence signals were detected using the multiplex array reader Luminex 200™ System (Luminex Corporation, Austin, TX, USA), and the data were analyzed.

### 2.17 Detection of adipokines

The Human Adipokine Array Kit (ARY024 from R&D Systems) was used according to the manufacturer’s instructions. Tissue lysates were prepared. Briefly, the membranes containing adipokine antibodies were blocked and then incubated with tissue lysates (50 µg) for 2 h at room temperature, washed in Wash Buffer, and then incubated with biotin‐conjugated antibodies for 2 h and with a horseradish peroxidase‐linked secondary antibody for another 2 h. The membranes were incubated with chemiluminescent substrate. The chemiluminescence was detected by ChemiDoc XRS System (Bio‐Rad, Hercules, CA, USA).

### 2.18 Apoptosis assay

Cell death of DLD1 and HCT116 cells induced by 3‐bromopyruvate (3‐BrPA) (Selleck Chemicals, Houston, TX, USA) was analyzed with flow cytometry using annexin V/PI assays (Keygen Company, Nanjing, China) according to the manufacturer's instructions. The cells were finally subjected to Beckman Coulter CytoFLEX flow cytometry, and results were analyzed using CellQuest Pro software.

### 2.19 Statistical analysis

Data were analyzed by either Student’s t‐test or one‐way ANOVA using the GraphPad Prism analysis software (Prism GraphPad 7.0). The results are expressed as the mean ± SD, and a P value < 0.05 was considered to be statistically significant.

## 3 Results

### 3.1 The expression of CPT1A is downregulated in CRC with peritoneal metastasis and correlates with poor prognosis

**Fig. 1:**
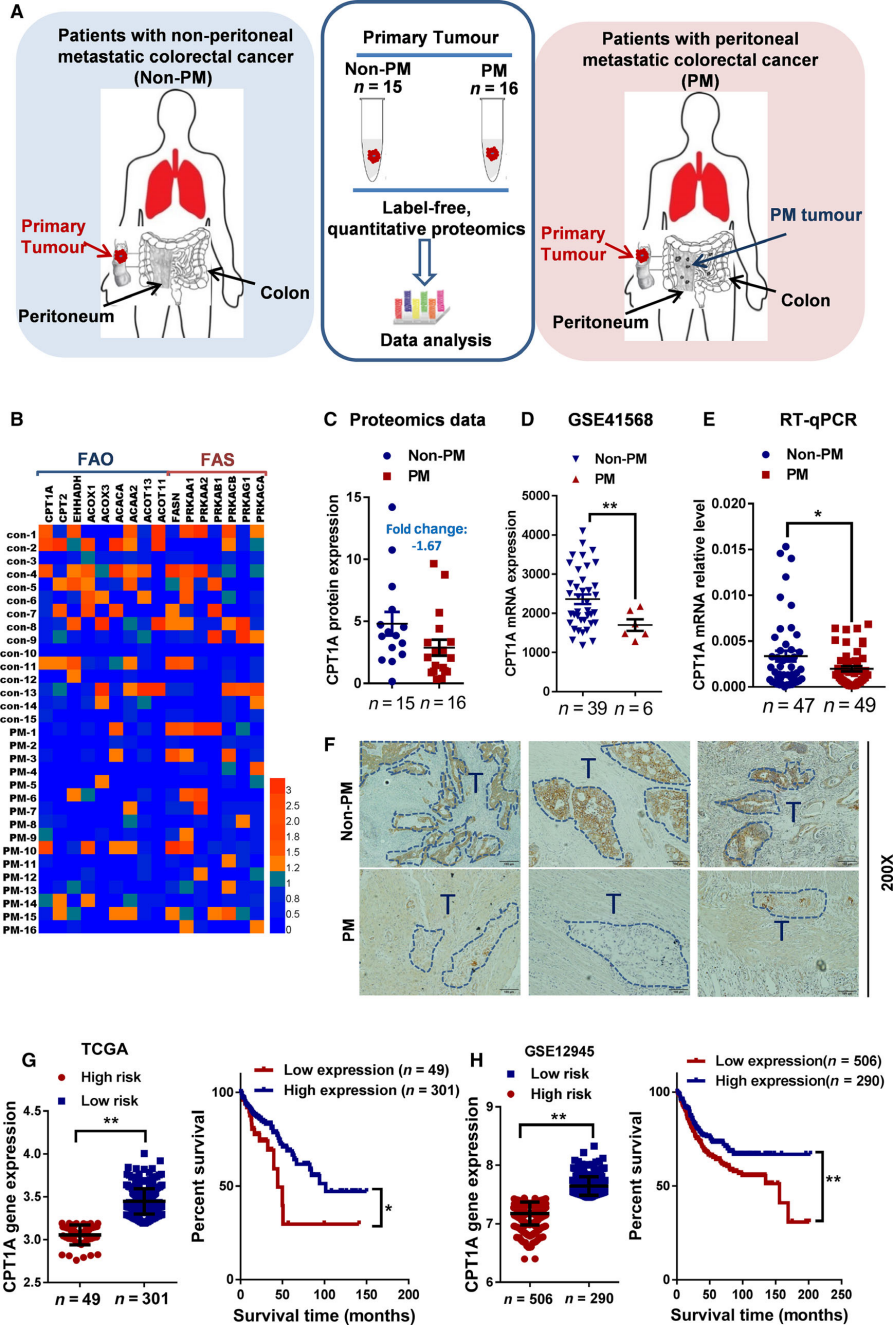
CPT1A is expressed at low levels in colon cancer patients with peritoneal metastasis and is associated with poor prognosis. (A) Schematic of label‐free, quantitative proteomics strategy in primary tumors from patients with nonperitoneal metastatic colorectal cancer (non‐PM) or patients with peritoneal metastatic colorectal cancer (PM). (B) Expression heatmap of 16 enzymes selected for FAO (fatty acid oxidation) and FAS (fatty acid synthase) pathways. Scales in log2‐normalized gene counts. (C) CPT1A expression in non‐PM (*n* = 15) and PM (*n* = 16) primary tumors derived from label‐free, quantitative proteomics (Student’s *t*‐test). (D) Scatter plots show the relative transcript abundance of CPT1A within patient samples from gene expression omnibus (GEO) PM and non‐PM colon cancer cohorts (Student’s *t*‐test). (E) qRT‐PCR for CPT1A in primary tumors from patients with non‐PM or PM (Student’s *t*‐test). (F) Immunohistochemical staining to detect CPT1A expression in primary tumors from patients with non‐PM or PM. (G) Kaplan–Meier curves showing survival for patients from TCGA cohorts partitioned by the relative abundance of CPT1A (high risk/low expression and low risk/high expression)(Student’s *t*‐test and Kaplan–Meier). (H) Kaplan–Meier curves showing survival for patients from the GEO (GSE12945) cohort partitioned by the relative abundance of CPT1A (high risk/low expression and low risk/high expression) (Student’s *t*‐test and Kaplan–Meier). Bars, mean ± SD. *P < 0.05, **P < 0.01.

Apart from the best‐known metabolic abnormality in cancer cells, aerobic glycolysis (also called the Warburg effect), there is compelling evidence showing that certain cancer cells/tissues, especially in an adipocyte‐rich environment, can also utilize fatty acids to generate energy to promote cancer cell growth and survival [6, 7, 8, 18]. To understand whether lipid metabolism is dysregulated in patients with peritoneal metastasis from colorectal cancer (PM‐CRC), 31 primary tumors with PM (n = 16) or non‐PM (n = 15) were selected, and quantitative proteomic analysis was performed to reveal the differentially expressed proteins (Fig. 1A). The expression levels of 16 proteins involved in regulating lipid metabolism are shown (Fig. 1B; Table S1). The key enzymes of the lipid synthesis pathway had no significant change, while the enzymes of fatty acid oxidation (FAO) and free fatty acid (FFA) transporter‐related pathways showed a decline trend in the PM‐CRC group compared with those in the control group.

FAO can catabolize FA and produce more than threefold as much ATP per mole as carbohydrate oxidation to fuel tumor proliferation in response to metabolic stress [19, 20]. Carnitine palmitoyltransferase I (CPTI), as the key rate‐limiting enzyme of FAO, controls FAO directly and thus facilitates the entry of FFAs into the mitochondria by loading fatty acyl groups onto carnitine [20, 21]. CPT1A expression was found to be closely related to a poor prognosis in prostate cancer, ovarian cancer, Burkitt’s lymphoma, glioblastoma, leukemia, breast cancer, and gastric cancer [20]. However, interestingly, the CPT1A protein was downregulated approximately by 1.67‐fold in PM‐CRC (Fig. 1C; Table S1). Importantly, the low expression of CPT1A in PM‐CRC was further substantiated by analysis of the GEO dataset (GSE41568, Fig. 1D). Analysis of Oncomine data revealed an obvious decrease in CPT1A mRNA expression in colorectal cancer compared with that in normal tissues (Table S2). In addition, a significant decrease in CPT1A expression was observed in clinical samples from PM‐CRC patients compared with that in the non‐PM patient samples by RT‐qPCR (Fig. 1E). The results of immunohistochemistry further demonstrated that the expression of CPT1A in tumor sites from PM‐CRC was lower compared with those from non‐PM‐CRC patients (Fig. 1F). To further confirm this relationship, we analyzed CPT1A expression and the survival probability of CRC from The Cancer Genome Atlas (TCGA) and the Gene Expression Omnibus (GEO) database, respectively. A statistically significant positive correlation was detected between low CPT1A expression and high risk of patients, and reduced CPT1A expression was significantly associated with shorter overall survival of patients from TCGA and GSE12945 datasets (Fig. 1G and H). These data demonstrated that decreased expression of CPT1A was associated with PM‐CRC and poor prognosis in CRC patients.

### 3.2 Low expression of CPT1A induces a metabolic shift toward glycolysis in CRC cells

**Fig. 2:**
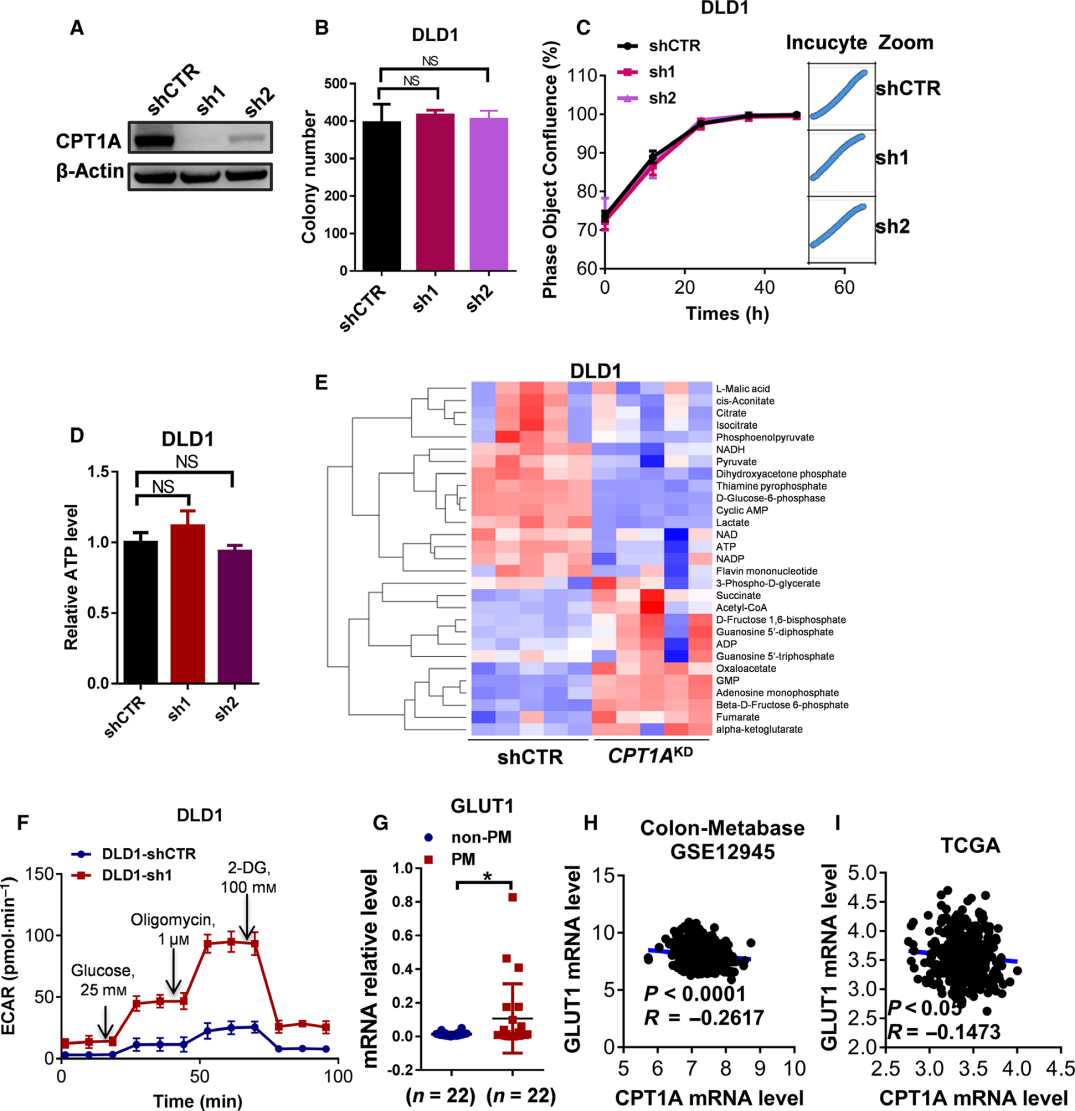
Low expression of CPT1A induces metabolic shift toward glycolysis in CRC cells. (A) Western blot analysis of CPT1A expression in *CPT1A* ^KD^ DLD1 cells. (B) The number of colonies in *CPT1A* ^KD^ DLD1 cells (one‐way ANOVA). (C) The proliferation rate of CPT1A^KD^ DLD1 cells evaluated by phase object confluence (%) with the IncuCyte ZOOM (one‐way ANOVA). Image data for phase object confluence processed by IncuCyte ZOOM software (Right). (D) Quantitative analysis of total ATP generation in *CPT1A* ^KD^ DLD1 cells (one‐way ANOVA). (E) Heatmap of metabolites in the energy pathway detected by Metabolomics in *CPT1A* ^KD^ DLD1 cells. The main discriminant metabolites are shown in Table S3. (F) Pharmacological profile of ECAR monitored with a Seahorse XF24 analyzer for 100 min in *CPT1A* ^KD^ DLD1 cells. The metabolic inhibitors glucose, oligomycin A, and 2‐DG were injected sequentially at different time points as indicated. (G) qRT‐PCR for GLUT1 in primary tumors from patients with non‐PM or PM (Student’s *t*‐test). (H and I) Correlation analysis of the mRNA levels of GLUT1 and CPT1A in CRC patients from the GSE12945 (H) and TCGA (I) datasets (Pearson's correlation analysis). Bars, mean ± SD. *P < 0.05, **P < 0.01.

Low expression of CPT1A is associated with poor prognosis in CRC patients, and CPT1A is expressed at low levels in patients with PM‐CRC. To better understand the metabolic status of CRC cells with low CPT1A expression, a lentiviral vector was constructed to silence the CPT1A gene in HCT116 and DLD1 cells (Fig. 2A; Fig. S1A). Our surprising results showed that *CPT1A* ^KD^ did not suppress colony formation or proliferation or affect the total levels of cellular ATP in CRC cells (Fig. 2B–D and Fig. S1B‐F). In addition, the protein expression levels of the AMPK system showed no significant change (P > 0.05) between PM‐CRC and non‐PM‐CRC primary tumors by quantitative proteomics, which also indicated that the energy balance was not disturbed (Fig. S2). Together, these results implied that other energy metabolism pathways are upregulated that compensate for inhibition of FAO is a possible mechanism.

Next, the energy metabolism‐related metabolites were tested by NMR‐based metabonomics for *CPT1A* ^KD^ DLD1 cells. 29 metabolites were covered in this metabolomics analysis. As shown in Fig. 2E and Table S3, the metabolites involved in glycolysis were significantly upregulated. Conversely, the majority of the metabolites in the tricarboxylic acid cycle were drastically decreased. Further, we also demonstrated that the reduction in CPT1A expression in HCT116 and DLD1 cells was paralleled by an increase in glycolysis, glycolytic reserve, and glycolytic capacity (Fig. 2F; Fig. S1G). Moreover, the mRNA level of GLUT1, a glucose transporter that meditates the first rate‐limiting step in glucose metabolism, was elevated in CRC‐PM by qRT‐PCR (Fig. 2G). Based on TCGA and GEO database analyses, the mRNA level of GLUT1 was negatively correlated with the enzyme CPT1A, which is essential for FAO (Fig. 2H and I). These data provide compelling evidence that PM‐CRC generally relies mostly on glycolysis rather than FAO. This finding is consistent with the point of view that increased glycolysis plays an essential role in the invasive phenotype of malignant cancers [22].

### 3.3 Increased CPT1A expression and FAO in PM‐CRC CAFs

**Fig. 3:**
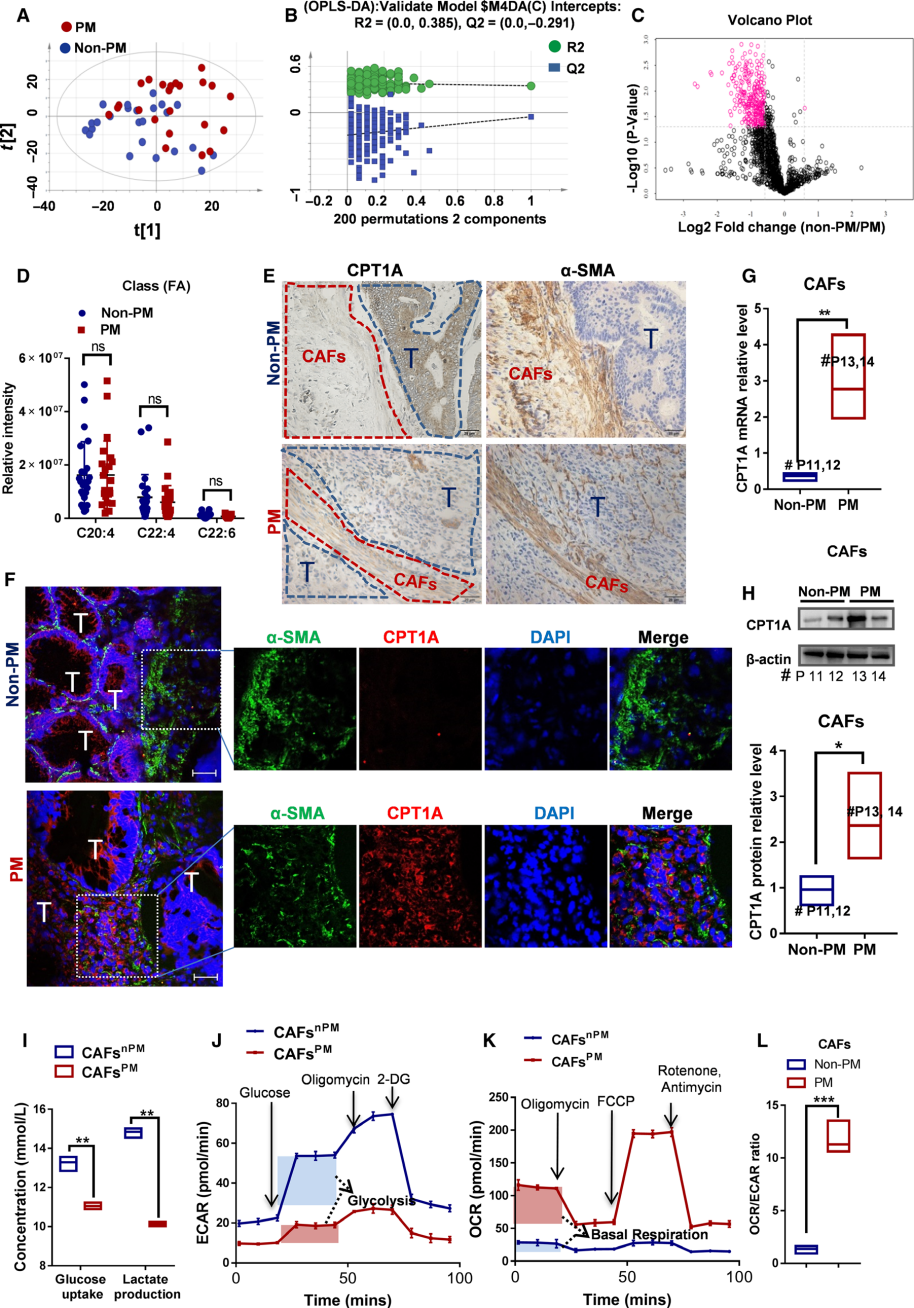
Cell metabolism switches to FAO by upregulating CPT1A in CAFs from patients with peritoneal metastasis. (A) Score plots are shown for PM (red) versus non‐PM (blue) from the orthogonal partial least squares discriminant analysis (OPLS‐DA) model. (B) Permutation is shown for PM versus non‐PM from the OPLS‐DA model. (C) A total of 1678 lipid species involved in cell metabolism are depicted in a volcano plot. The main discriminant metabolites are shown as pink cycles. (D) Measurements of FFA in primary tumors from patients with non‐PM or PM (Student’s *t*‐test). (E) The serial sections and immunohistochemical for CPT1A and α‐SMA in tumor tissues. 'T' (*blue areas*) indicates the tumor cells, and CAFs (*red areas*) were identified by α‐SMA. (F) The expression of CPT1A in CAFs from patients by immunofluorescence. Tumors of frozen colorectum sections were costained with CPT1A (red) and α‐SMA (green) antibodies and DAPI (blue), scale bar is 50 µm. Representative images of immunofluorescence staining in tumors from each group. (G) Expression of CPT1A (qRT‐PCR) in CAFs isolated from colon cancer samples with PM or non‐PM (Student’s *t*‐test). (H) Expression of CPT1A (western blot) in CAFs isolated from colon cancer samples with PM or non‐PM (Student’s *t*‐test). (I) Relative levels (% of control) of glucose uptake and lactate production in CAF^PM^ cells compared with CAF^nPM^ cells (Student’s *t*‐test). (J) The extracellular acidification rate (ECAR) was monitored with a Seahorse XF24 analyzer for 100 min. The metabolic inhibitors glucose, oligomycin A, and 2‐DG were injected sequentially at different time points as indicated. (K) The oxygen consumption rate (OCR) was monitored with a Seahorse XF24 analyzer for 100 min. The metabolic inhibitors oligomycin, FCCP, rotenone, and antimycin were injected sequentially at different time points as indicated. (L) OCR/ECAR ratios (Student’s *t*‐test). ECAR measurement equation used for glycolysis; OCR measurement equation used for basal respiration. Bars, mean ± SD. *P < 0.05, **P < 0.01, ***P < 0.001, *n* = 3. ns, not significant.

Given that the key enzymes of the lipid metabolism pathway were decreased in PM‐CRC, we detected the lipid flux in tumor tissues from patients with either PM or non‐PM by lipidomics. The lipid species of significant differences were screened with OPLS‐DA model of VIP > 1 and P value < 0.05 as screening criteria, of which a total of 42 lipid species were significantly changed (Fig. 3A–C). Although the enzymes of the FAO and FFA transporter‐related pathways were decreased in the tumors from PM‐CRC, no difference was found in FFA levels in the tumor microenvironment (TME) (Fig. 3D). A possible explanation for this seemingly contradictory result may be that some other cell types within the distinctive TME, including fibroblasts, mesothelial cells, immune cells, adipocytes, and endothelial cells, alter the metabolism to obtain FFAs, which contributes to cancer progression, invasion, and metastasis. The TME contains multiple cells and cytokines, which play an important role in tumor proliferation, invasion, and metastasis [23], and has received widespread attention in recent years [24]. Among all the stromal cells that populate the TME, cancer‐associated fibroblasts (CAFs) are the most abundant and essential for cancer pathogenesis, progression, and metastasis [23, 24, 25]. We hypothesized that CAFs could experience an increase in FAO by upregulating CPT1A expression to increase lipid oxidation resulting from tumor‐imposed glucose restriction. CAFs isolated from fresh human colon cancer samples were characterized by positive expression of α‐smooth muscle actin (α‐SMA) (Fig. S3A). The distribution of CPT1A in tumor cells and CAFs by serial immunohistochemistry indicated that CPT1A was upregulated in CAFs^PM^ than CAFs^nPM^ which was identified by the a‐SMA + cell coverage (red areas), whereas expression of CPT1A was significantly lower in PM‐CRC tumor cells, compared with those in non‐PM‐CRC tumor cells(Fig. 3E and Fig. S3B). Further, immunofluorescence staining showed that CPT1A expression was indeed increased in CAFs^PM^ compared with CAFs in non‐PM‐CRC tissue (CAFs^nPM^) (Fig. 3F). Consistently, the CAFs isolated from fresh PM‐CRC samples also exhibited an increased expression of the FAO‐related enzyme CPT1A (Fig. 3G and H). To determine whether these effects were specific to metabolic reprogramming and a metabolic switch from glycolysis to FAO in CAFs^PM^, we measured glucose uptake and lactate production in CAFs^nPM^ and CAFs^PM^ by biosensor. Glucose uptake and lactate production measurements, obtained at 24 h, showed that both were decreased in CAFs^PM^ (Fig. 3I). To further detail the metabolic alterations in CAFs^PM^, a Seahorse assay was performed to measure glycolysis and mitochondria oxygen consumption. CAFs^PM^ showed decreased extracellular acidification rate (ECAR; Fig. 3J) and increased oxygen consumption rate (OCR; Fig. 3K). Moreover, the ratio of OCR/ECAR clearly demonstrated a preferential increase in OCR, which confirmed the metabolic reprogramming toward FAO (Fig. 3L). These data implied that CAFs^PM^ underwent a shift in metabolism from glycolysis to fatty acid oxidation for energy supply.

### 3.4 Upregulation of CPT1A in CAFs promotes CRC cell migration and invasion, especially under low‐glucose conditions

**Fig. 4:**
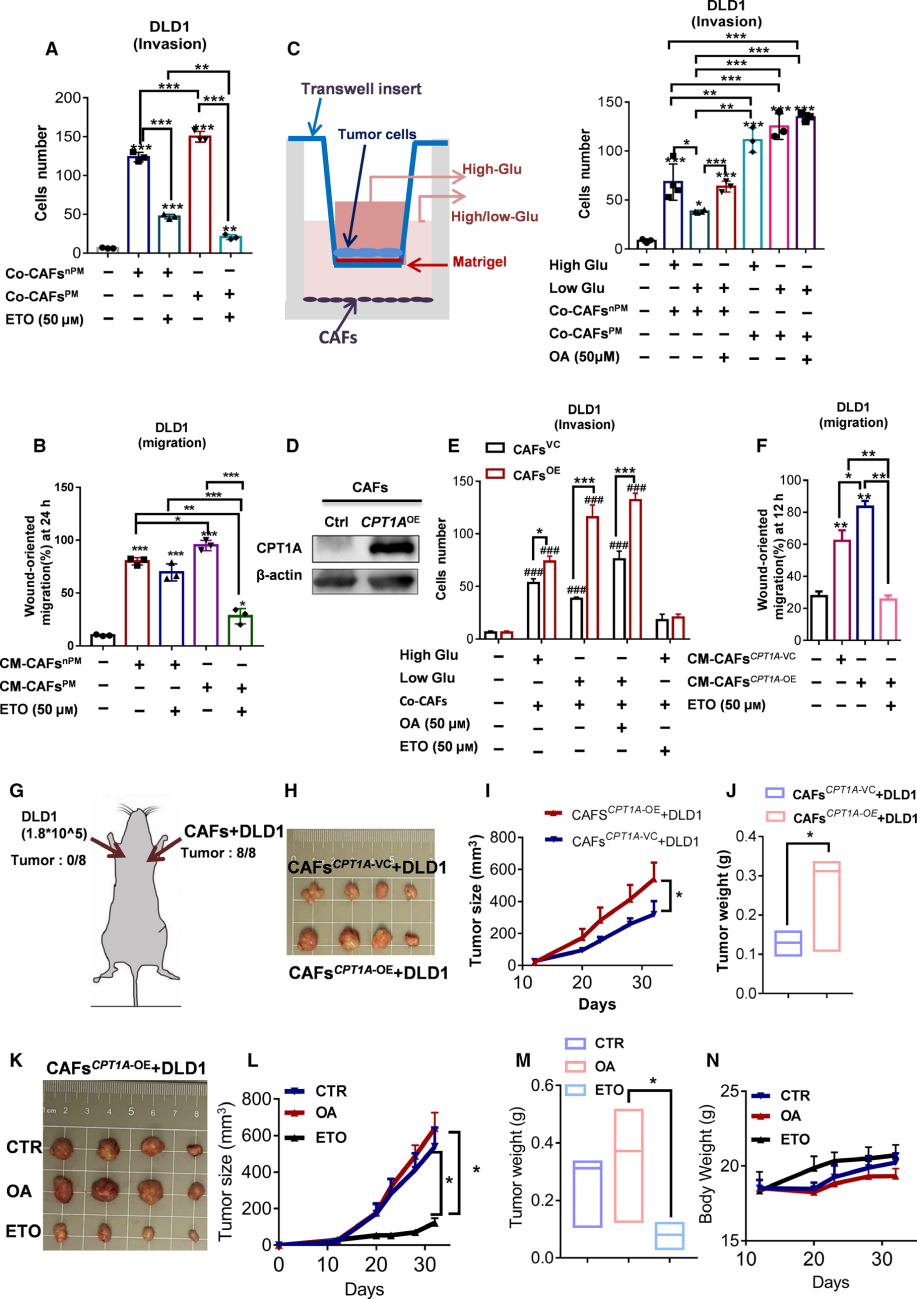
Upregulation of CPT1A in CAFs promotes CRC cell migration, invasion, and growth. (A) The cell number of crystal violet staining to quantify DLD1 cell Transwell invasion after 24 h of exposure to CAFs^PM^ or CAFs^nPM^ and ETO treatment (one‐way ANOVA), *n* = 3. (B) The wound healing ability of DLD1 incubated with CM from CAFs^PM^ or CAFs^nPM^ and ETO treatment detected by IncuCyte ZOOM (one‐way ANOVA), *n* = 3. (C) Schematic of CRC cells cocultured with CAFs under high/low‐glucose conditions (left panel). The cell number of crystal violet staining to quantify DLD1 cell Transwell invasion after 24 h of exposure to CAFs^PM^ or CAFs^nPM^ under high/low‐glucose conditions (right panel) (one‐way ANOVA), *n* = 3. (D) CAFs stably expressing high levels of CPT1A (western blot) established by lentivirus transfection. (E) The cell number of crystal violet staining to quantify DLD1 cell Transwell invasion after 24 h of exposure to CAFs*^CPT1A‐^* ^VC^ or CAFs*^CPT1A‐^* ^OE^. # means compared with the corresponding control groups (one‐way ANOVA), *n* = 3. (F) The wound healing ability of DLD1 cells incubated with CM from CAFs*^CPT1A‐^* ^VC^ or CAFs*^CPT1A‐^* ^OE^ detected by IncuCyte ZOOM(one‐way ANOVA), *n* = 3. (G) Injection sites in the xenograft mouse model. (H) Representative tumors from the indicated groups, *n* = 4 mice per group. (I) Tumor growth of DLD1 cells coinjected with CAFs*^CPT1A‐^* ^VC^ or CAFs*^CPT1A‐^* ^OE^(two‐way ANOVA), *n* = 4 mice per group. (J) Tumor weight of DLD1 cells coinjected with CAFs*^CPT1A‐^* ^VC^ or CAFs*^CPT1A‐^* ^OE^(Student’s *t*‐test), *n* = 4 mice per group. (K) Representative tumors from the indicated groups, *n* = 4 mice per group. (L) Tumor growth of DLD1 cells coinjected with CAFs*^CPT1A‐^* ^OE^ after treatment with OA or ETO, *n* = 4 mice per group (one‐way ANOVA). (M) Tumor weight from mice coinjected with DLD1 cells and CAFs*^CPT1A‐^* ^OE^ after treatment with OA or ETO, *n* = 4 mice per group (one‐way ANOVA). (N) Body weight of mice coinjected with DLD1 cells and CAFs*^CPT1A‐^* ^OE^ after treatment with OA or ETO (one‐way ANOVA), *n* = 4 mice per group. Bars, mean ± SD. *P < 0.05, **P < 0.01, ***P < 0.001.

Then, to determine whether CAFs^PM^ can increase tumor cell migration and invasion, the Transwell assay was carried out. Both CAFs^nPM^ and CAFs^PM^ significantly increased the migration and invasion of HCT116 and DLD1 cells compared with those in the control, but CAFs^PM^ had a more powerful effect on tumor metastasis (Fig. 4A; Fig. S4A and B). Consequently, CAFs^PM^ were more sensitive to the CPT1 inhibitor etomoxir (ETO) (Fig. 4A; Fig. S4A and B). Consistently, the wound migration test showed that CM from CAFs^nPM^ or CAFs^PM^ accelerated the cell migration and invasion ability of HCT116 and DLD1 cells, but CM from CAFs^PM^ treated with ETO significantly reduced the migration of CRC cells (Fig. 4B; Fig. S5).

Tumor cells upregulate glycolysis, resulting in increased glucose consumption, which may lead to glucose depletion within the TME [22, 26]. Metabolic shifting such as glucose deprivation in the TME impairs the antitumor effects of CD8 + T cells [27, 28]. Thus, we next explored the metabolic reprogramming in CAFs cells from TME of PM‐CRC. By coculturing CAFs^nPM^ and CAFs^PM^ with CRC cells under high‐glucose medium (4.5 g·L^−1^ glucose) or low‐glucose medium (1 g·L^−1^ glucose), we found that CAFs^nPM^ significantly repressed the migration and invasion of HCT116 and DLD1 cells in glucose‐low media, but this effect was rescued by supplying FFAs (oleic acid, OA) (Fig. 4C; Fig. S4C and D). The opposite results were observed with CAFs^PM^, which promote cell migration and invasion in glucose‐low media (Fig. 4C; Fig. S4C and D). These results indicated that if the metabolic balance between tumors and CAFs is perturbed, CAFs^PM^ might be able to respond to FFA readily. The switch from glucose to FFA oxidation that occurs in the CAFs^PM^ via upregulation of CPT1A expression has an important role in promoting CRC cell migration and invasion. To confirm that upregulation of CPT1A expression in CAFs could increase CRC cell migration and invasion, CAFs stably expressing CPT1A were first constructed via a lentivirus system (Fig. 4D). The cell proliferation rate was monitored with the IncuCyte ZOOM live cell imager. An 84‐h‐long cell proliferation assay indicated that both cell types, CAFs*^CPT1A‐^* ^VC^ and CAFs*^CPT1A‐^* ^OE^, exhibited a uniform pattern of growth under high‐glucose conditions (Fig. S6A). When cultures were deprived of most glucose, the CAFs*^CPT1A‐^* ^VC^ exhibited a significant delay in cell growth compared with CAFs*^CPT1A‐^* ^OE^ (Fig. S6B). Thus, our evaluation of cell proliferation appears to validate the results that the growth and proliferation of CAFs*^CPT1A‐^* ^VC^ rely more on glucose, but low glucose induces a shift in energy metabolism from glucose to FA in CAFs*^CPT1A‐^* ^OE^. Consistently, the Transwell invasion test showed that CAFs*^CPT1A‐^* ^OE^ accelerated cell invasion (Fig. 4E; Fig. S6C‐E) and increased the wound healing ability of DLD1 and HCT116 when cultures were deprived of glucose, but directly blocking FAO in CAFs*^CPT1A^* ^‐OE^ with etomoxir(ETO) inhibits migration and invasion (Fig. 4F; Fig. S6 F‐H).

To determine whether CAFs promote the formation and growth of colorectal cancer *in vivo*, we injected DLD1 cells alone or mixed with CAFs into the left and right sides of nude mice, respectively; this injection protocol eliminates variation among mice to ensure comparable results in different groups (Fig. 4G). As expected, DLD1 did not form tumors *in vivo* (only 1.8*10^5^ cells per mouse), while CAFs played an important role in tumorigenesis and greatly enhanced the growth of tumor cells *in vivo* (Fig. 4G‐J). When DLD1 cells mixed with CAFs*^CPT1A‐^* ^VC^ or with CAFs*^CPT1A‐^* ^OE^ were injected using the same protocol above, we found that DLD1 cells mixed with CAFs*^CPT1A‐^* ^OE^ grew faster than DLD1 cells mixed with CAFs*^CPT1A‐^* ^VC^ (Fig. 4H‐J). To investigate whether the targeting of CPT1A is responsible for the tumor‐inhibiting effects of CAFs*^CPT1A‐^* ^OE^ *in vivo*, a CPT1 inhibitor was used to elucidate the function of CAFs. Tumors obtained from the CAF*^CPT1A‐^* ^OE^‐treated ETO (40 mg·kg^−1^) group showed significantly smaller tumor growth compared with the tumors from the control group (Fig. 4K‐M), and the therapeutic effect was well tolerated with the increase in body weight (Fig. 4N). In contrast, the transplanted tumors grew to a larger size when fed 1% OA in drinking water, although the difference was not statistically significant (Fig. 4K‐M). Similarly, CM from CPT1A‐upregulated CAFs (CAFs^PM^/CAFs*^CPT1A‐^* ^OE^) induced the proliferation and growth of CRC cells *in vitro* (Fig. S7). These data demonstrated that the upregulation of CPT1A in CAFs can promote tumor growth rapidly and that FA catabolism is required to maintain the function and proliferation of CAFs^PM^ when access to glucose is limited.

### 3.5 CPT1A overexpression in CAFs leads to FAO impairment associated with a metabolic shift toward aerobic glycolysis in colon cancer cells

**Fig. 5:**
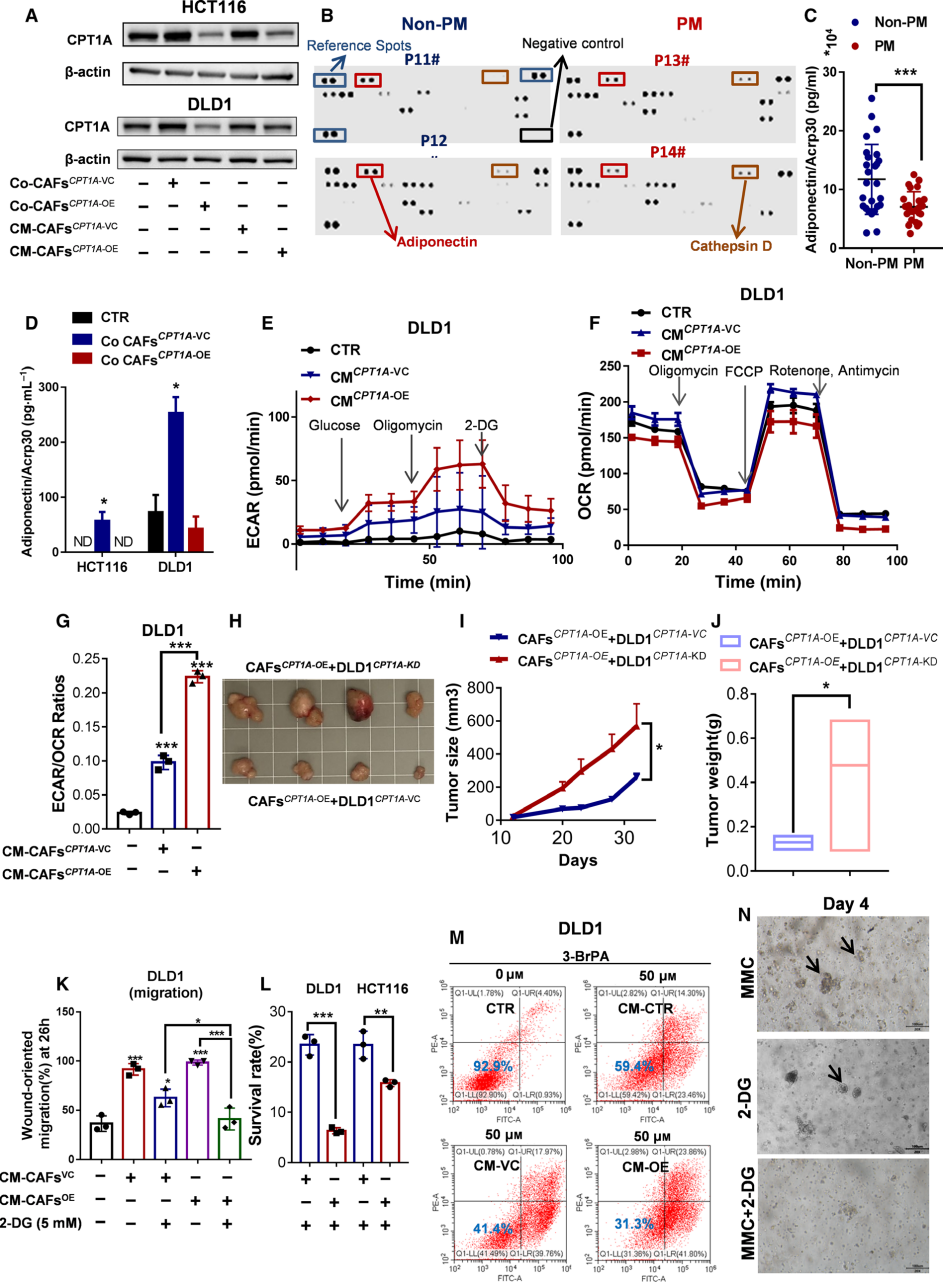
Glycolysis compensates for FAO loss in CRC cells and were sensitive to glycolysis inhibitor 2‐DG. (A) Western blot showing CPT1A expression in DLD1/HCT116 cells cocultured with CAFs*^CPT1A‐^* ^VC^ or CAFs*^CPT1A‐^* ^OE^ separated by a Transwell cell culture insert or in the CM under high‐glucose conditions. (B) The Human Adipokine Array detects multiple adipokines in primary tumor tissue lysates from patients with PM or non‐PM. (C and D) Luminex magnetic bead‐based suspension array was used for adiponectin/Acrp30 quantification in primary tumor tissue lysates from patients with PM or non‐PM (C) (Student’s *t*‐test) and in supernatant medium from CRC cells cocultured with CAFs (D) (one‐way ANOVA). (E) ECAR and (F) OCR were measured under basal conditions and after the addition of the indicated drugs to DLD1 cells by a Seahorse XF24 analyzer, *n* = 3. (G) ECAR/OCR ratios (one‐way ANOVA). ECAR measurement equation used for glycolysis; OCR measurement equation used for basal respiration. (H) Tumor images of DLD1*^CPT1A‐^* ^VC^ or DLD1*^CPT1A‐^* ^KD^ cells coinjected with CAFs*^CPT1A‐^* ^OE^, *n* = 4 mice per group. (I) Tumor growth of DLD1*^CPT1A‐^* ^VC^ or DLD1*^CPT1A‐^* ^KD^ cells coinjected with CAFs*^CPT1A‐^* ^OE^(two‐way ANOVA), *n* = 4 mice per group. (J) Tumor weight of DLD1*^CPT1A‐^* ^VC^ or DLD1*^CPT1A‐^* ^KD^ cells coinjected with CAFs*^CPT1A‐^* ^OE^(Student’s *t*‐test), *n* = 4 mice per group. (K) The wound healing ability of DLD1 (left panel) and HCT116 (right panel) cells incubated with CM (conditioned medium) from CAFs*^CPT1A‐^* ^VC^ or CAFs*^CPT1A‐^* ^OE^ and then treated with 2‐DG by IncuCyte ZOOM (one‐way ANOVA), *n* = 3. (L) The survival rate of DLD1/HCT116 cells evaluated by CCK‐8 after incubation with CM (conditioned medium) from CAFs*^CPT1A‐^* ^VC^ or CAFs*^CPT1A‐^* ^OE^ and treatment with 2‐DG (5 mm) for 72 h (Student’s *t*‐test), *n* = 3. (M) Cell viability of DLD1 cells was assessed by annexin V/PI assay after 48‐h treatment with 3‐BrPA. The value in each panel indicates the % of survival cells. (N) Morphological comparison of the therapeutic activity of the glycolysis inhibitor 2‐DG combined with mitomycin C in organoids derived from CRC‐PM (bright field, scale bar 100 μm), Bars, mean ± SD. *P < 0.05, **P < 0.01, ***P < 0.001, *n* = 3.

Recent findings suggest that stromal cells in the TME regulate metabolism in adjacent cancer cells [29, 30]. Thus, we wanted to determine whether the reduced level of CPT1A in CRC tumors is caused by CAFs with high CPT1A expression. To test this hypothesis, we incubated the CAFs*^CPT1A‐^* ^VC^/CAFs*^CPT1A‐^* ^OE^, separated by a Transwell insert, with DLD1 and HCT116 cells or incubated them with CM. After coculture or incubation for 48 h, CPT1A protein expression in CRC cells was detected by western blotting. As shown in Fig. 5A, CAFs*^CPT1A‐^* ^OE^ significantly decreased the level of CPT1A in tumor cells; in contrast, the expression of CPT1A in CRC cells can be upregulated by vector control CAFs. However, when the tumor cells were exposed to low glucose, the expression of CPT1A did not decrease but slightly increased (Fig. S8A), which was consistent with reports that CPTI activates FAO to increase ATP and NADPH reserves, and protects cancer from environmental stress such as glucose deprivation and hypoxia [31].

Adipokines are linked to metabolic dysfunction, insulin resistance, and inferior outcomes in cancer treatment [32, 33]. To identify factors responsible for lipid metabolic reprogramming, we performed a human adipokine array (Fig. 5B). Compared with non‐PM tissues, we found that among 58 adipokines tested, the 2 adipokines that were most abnormally expressed in PM tissues were adiponectin and cathepsin D. It was consistent with reports that cathepsin D enhances cancer invasion and metastasis [34, 35, 36]. Adiponectin can directly influence fatty acid utilization, by regulating lipolysis, fatty acid transport, and β‐oxidation [37]. The Luminex bead‐based suspension array was performed in a 96‐well plate to detect the adiponectin level in CRC tumors. Congruently, the level of adiponectin was obviously low in PM tissues (Fig. 5C). To determine whether reducing adiponectin is associated with CPT1A expression, the cell culture supernatant of cells that were cocultured with CAFs was determined. As expected, the results showed that adiponectin expression was strongly upregulated in the cell culture supernatant from CAFs*^CPT1A‐^* ^VC^, whereas it was decreased after cocultivation with CAFs*^CPT1A‐^* ^OE^ (Fig. 5D). Taken together, these findings suggest that CAFs*^CPT1A‐^* ^OE^ can induce lower CPT1 expression by decreasing adiponectin secretion.

Next, we evaluated metabolic alterations in colon cancer cells after coculture with CAFs. It is a well‐known fact that tumors could meet increasing demands for rapidly available energy by upregulating glucose consumption and metabolism [6]. However, it is still unclear whether metabolic changes occur within tumor cells in the metabolically challenging tumor microenvironment. In our study, we showed that CAFs shift toward FFA oxidation as an energy source for growth and survival, but cancer cells decrease in FFAs uptake. Then, we examined the metabolic and functional consequences of CAF‐induced glucose metabolism in cancer cells. As expected, the CAFs*^CPT1A‐^* ^OE^ induced increase in glucose uptake and the metabolic shift toward the 'Warburg phenotype', which was confirmed by an increase in the glycolytic capacity and glycolytic reserve (Fig. 5E, G; Fig. S8B and D), and a decrease in oxidative phosphorylation (Fig. 5F; Fig. S8C). These data suggested that the adaptation of glucose metabolism allows colon cancer cells to thrive on glucose acquired from the surrounding environment. To further study CAF*^CPT1A‐^* ^OE^ cells' role in promoting tumor with lower CPT1A expression growth in vivo, a tumor‐bearing mouse model was established. DLD1 cells with CPT1A knockdown (*CPT1A* ^KD^ DLD1) were mixed with CAFs and then injected subcutaneously into nude mice. *CPT1A* ^KD^ DLD1 cells that contained CAFs*^CPT1A‐^* ^OE^ grew larger tumors than their corresponding controls (Fig. 5H‐J). Taken together, these data suggested that CAF*^CPT1A‐^* ^OE^ cells may provide a benefit to the tumor by promoting anaerobic glycolysis but conducting minimal oxidization of fatty acids (FAs) in CRC cells, which further implied that PM‐CRC tumors and CAFs maintain a perfect balance of energy metabolic.

Based on the observation that CRC cells cocultured with CAFs*^CPT1A‐^* ^OE^/CAFs^PM^ resulted in highly glycolytic, we speculated that this subpopulation might be sensitive to glycolytic inhibition. Therefore, we speculated that tumors in the PM group are more sensitive to glycolysis inhibitors. The glycolysis inhibitor 2‐deoxy‐D‐glucose (2‐DG),is used as a tumor therapeutic and evaluated in clinical trials [38]. We first tested whether 2‐DG could inhibit CRC cell growth after incubation with CM from CAFs*^CPT1A‐^* ^OE^ by disrupting their energy metabolism. Although no significant apoptosis occurred, 2‐DG at 5 mM significantly reduced CRC cell migration at 26h and growth at 72 h after incubation with CM from CAFs*^CPT1A‐^* ^OE^ compared with CAFs*^CPT1A‐^* ^VC^ (Fig. 5K and L; Fig. S8E and F; Fig. S9). Similarly, their apoptotic response to another inhibitor of glycolysis (3‐BrPA), a potent monotherapy against various tumors [39], was also significantly increased (Fig. 5M; Fig. S8H), indicating that CRC cells cocultured with CAFs*^CPT1A‐^* ^OE^ are more sensitive to glycolytic inhibition. To evaluate 2‐DG as a novel combination therapy for colorectal PM, tumor‐derived organoids were used. Three CRC patient‐derived PM‐organoid lines were successfully established for this study. Ryan et al showed that oxaliplatin‐based HIPEC is not very effective in the clinical setting [40], but mitomycin C(MMC) was an effective drug at clinical concentrations in human CRC‐PM‐organoid model. Then combination treatment with MMC and glycolysis inhibitor was assessed. The glycolysis inhibitor 2‐DG showed limited toxicity as a monotherapy but greatly sensitized all PM‐CRC organoids to MMC (Fig. 5N). These data together suggested that glycolysis inhibition is a feasible combination strategy for the treatment of patients with PM‐CRC.

### 3.6 Cytokines derived from CAFs that enhance FA metabolism could contribute to the proliferation, invasion, and metastasis of CRC

**Fig. 6:**
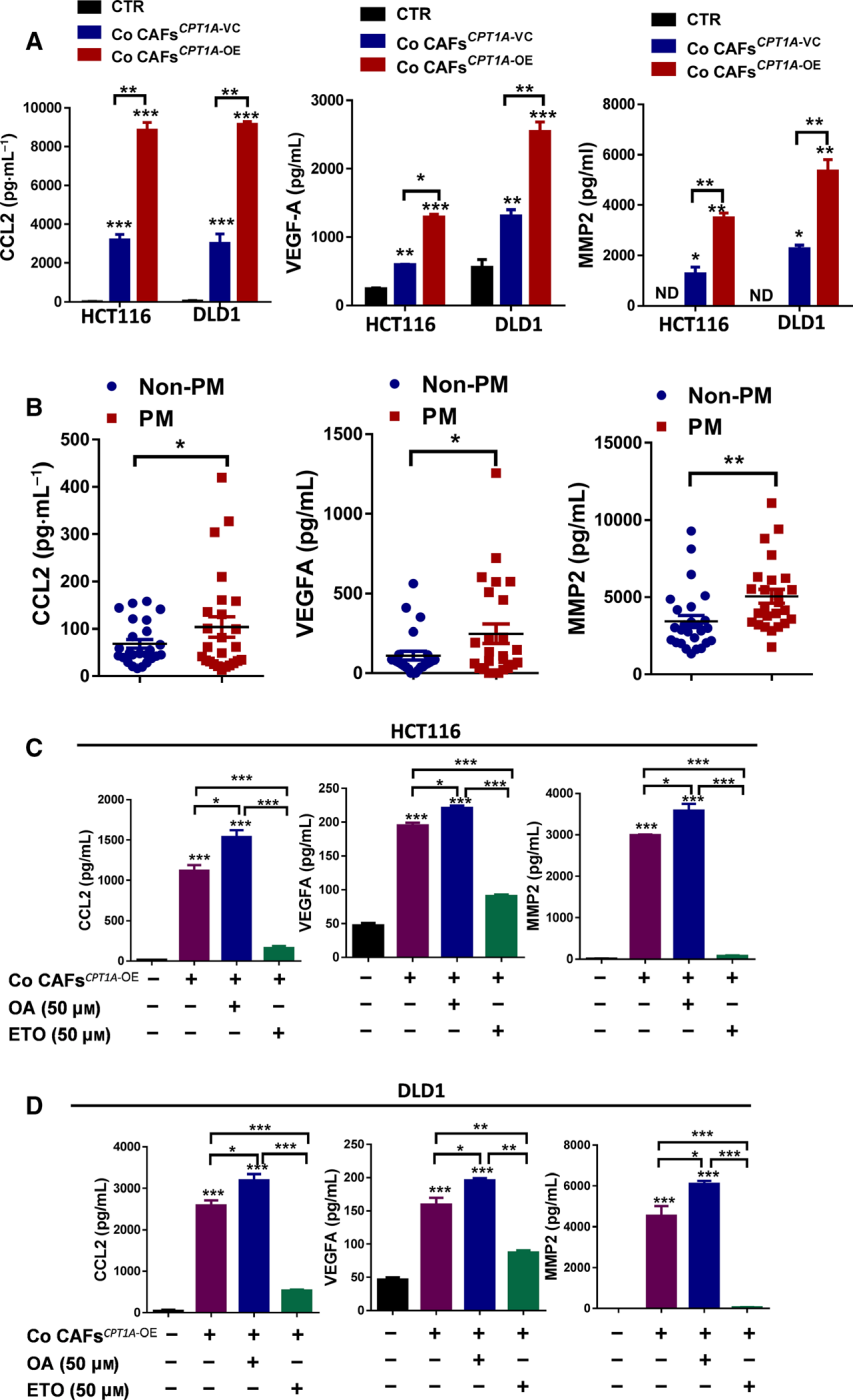
Cytokines derived from CAFs that enhance FA metabolism and may contribute to the proliferation, invasion, and metastasis of CRC. (A) The Luminex magnetic bead‐based suspension array was used for CCL2 (left panel), VEGF‐A (middle panel) and MMP2 (right panel) quantification in supernatant medium from CRC cells cocultured with CAFs (one‐way ANOVA). (B) The Luminex magnetic bead‐based suspension array was used for CCL2 (left panel), VEGF‐A (middle panel) and MMP2 (right panel) quantification in primary tumor tissue lysates from patients with PM or non‐PM (Student’s *t*‐test). (C) The Luminex magnetic bead‐based suspension array was used for CCL2 (left panel), VEGF‐A (middle panel), and MMP2 (right panel) quantification in supernatant medium from HCT116 cells cocultured with CAFs after the indicated drug treatment (one‐way ANOVA). (D) The Luminex magnetic bead‐based suspension array was used for CCL2 (left panel), VEGF‐A (middle panel), and MMP2 (right panel) quantification in supernatant medium from DLD1 cells cocultured with CAFs after the indicated drug treatment (one‐way ANOVA). Bars, mean ± SD. *P < 0.05, **P < 0.01, ***P < 0.001, *n* = 3.

It has been proven that glucose declines within the TME during tumor progression. In the TME, competition for common resources must exist between cancer cells and the surrounding other cells. Our study indicated that tumor cells seem to rely more on glycolysis, while CAFs from PM actively oxidize fatty acids (FAs) and conduct minimal glycolysis. To further assess the impact of FA catabolism on CAF cell functions, we used CPT1A‐overexpressing CAFs or etomoxir (ETO), an irreversible inhibitor of CPT1 that decreases mitochondrial FAO. Cytokines derived from CAFs were determined using the Human Magnetic Luminex Screening Assay. Among the 11 cytokines tested (CCL2, EGF, MIF, TNF‐a, IFN‐γ, IL‐2, IL‐10, IL‐12/23 p40, VEGF‐A, MMP2, and MMP9), the four cytokines abundantly secreted by CAFs after coculture with CRC cells were CCL2, IL‐12/23 p40, VEGF‐A, and MMP2. However, *in vitro* coculture of CRC cells with CAFs*^CPT1A‐^* ^OE^, compared with vector control CAFs, significantly increased CCL2, VEGF‐A, and MMP2 secretion (Fig. 6A). Congruently, all 3 cytokines were highly expressed in CRC tissues with peritoneal metastasis (Fig. 6B). In addition, the secretion of CCL2, VEGF‐A, and MMP2 was increased in OA‐treated CAFs stimulated in glucose‐low medium compared with non‐OA‐treated CAFs*^CPT1A‐^* ^OE^(Fig. 6C‐D). In contrast, ETO significantly decreased the secretion of CCL2, VEGF‐A, and MMP2 by CAFs*^CPT1A‐^* ^OE^ (Fig. 6C‐D). These data demonstrated that the function of CAF*^CPT1A‐^* ^OE^ cells was improved under low‐glucose but fatty acid‐rich conditions, which contribute to the proliferation, invasion, and metastasis of CRC cells.

### 3.7 Upregulation of CPT1A in CAFs is responsible for intraperitoneal tumor dissemination and growth

**Fig. 7:**
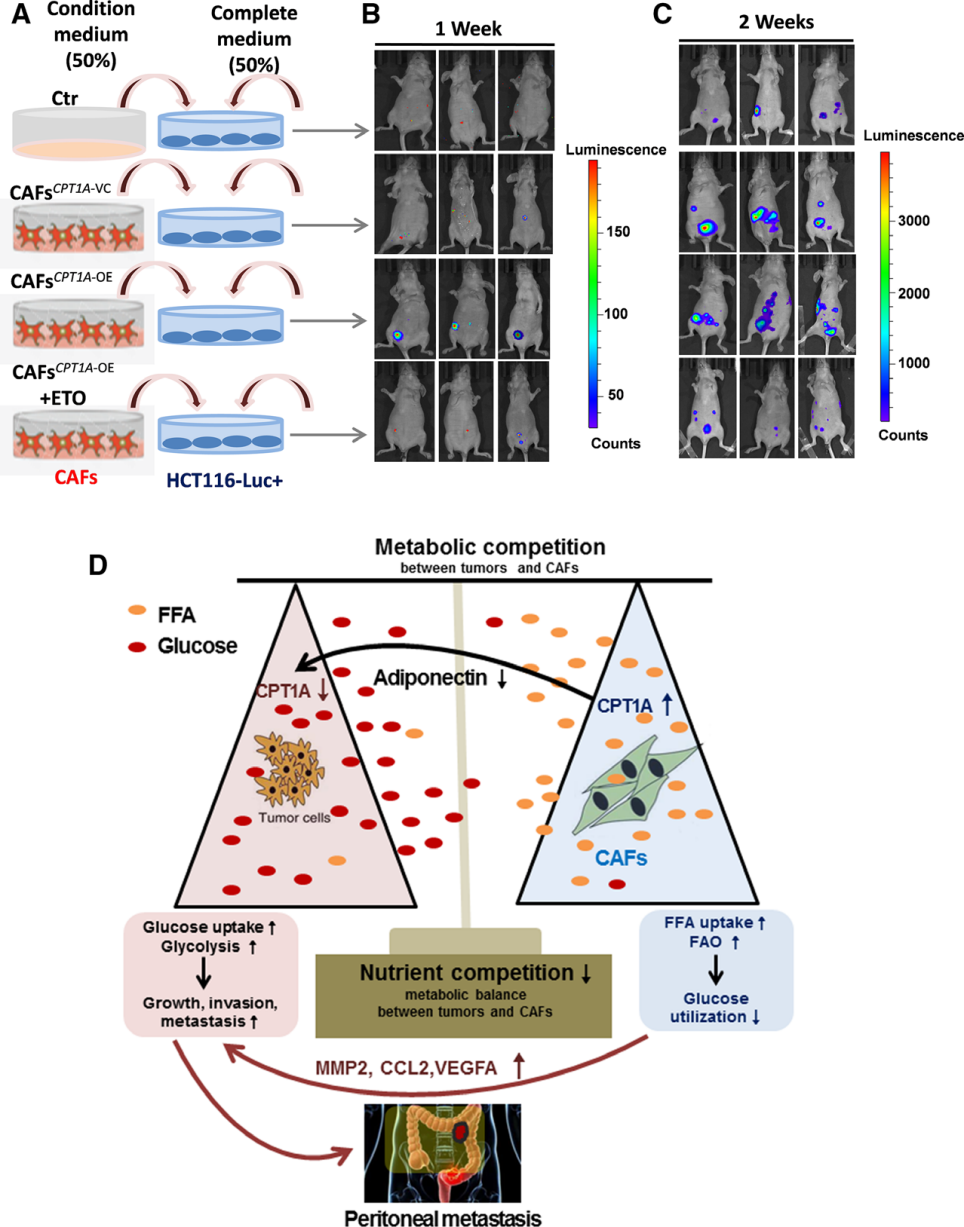
Upregulation of CPT1A in CAFs is responsible for intraperitoneal tumor dissemination and growth. (A) Experimental schema used to treat HCT116‐luc + cells before i.p. injection into BALB/C nude mice. (B) Animals from each test group were bioimaged (Xenogen IVIS system) by detecting the luciferase emission spectrum at 1 week post i.p. injection to visualize the progression of tumor growth in the peritoneum. (C) Animals from each test group were bioimaged (Xenogen IVIS system) by detecting the luciferase emission spectrum at 2 weeks post i.p. injection to visualize the progression of tumor growth in the peritoneum. (D) Summary of metabolic changes that occur in interacting colon cancer cells and CAFs as described in the text.

To investigate whether CAFs are responsible for peritoneal dissemination and growth of human colorectal cancer cells *in vivo*, we treated HCT116‐Luc+ (luciferase expression) cells with the indicated CM (Fig. 7A). Then, the cells were injected intraperitoneally (i.p.). Injection of HCT116‐Luc + cells after treatment with CAF‐CM resulted in the formation of tumors inside the abdominal cavity up to 7 days postinjection, especially in the CAFs*^CPT1A‐^* ^OE^‐CM group (Fig. 7B). This was followed by metastatic progression to other locations in the abdomen at 2 weeks postinjection. We identified that stimulation of cancer cells with CM from CAFs*^CPT1A‐^* ^VC^ or CAFs*^CPT1A‐^* ^OE^, especially CAFs*^CPT1A‐^* ^OE^, was able to induce tumor growth and intraperitoneal dissemination 2 weeks after i.p. injection (Fig. 7C). To explore whether pharmacological blockade of CPT1 reduced the function of CAFs in tumor growth and intraperitoneal dissemination, the CPT1 inhibitor etomoxir was used. As expected, etomoxir‐treated CM from CAFs*^CPT1A‐^* ^OE^ obviously reduced tumor growth and peritoneal dissemination (Fig. 7B‐C). These data together demonstrated that high expression of CPT1A in CAFs is responsible for intraperitoneal tumor dissemination and growth. The mechanistic model of our findings is presented in Fig. 7D.

## 4 Discussion

Peritoneal metastatic colorectal cancer leads to poor overall survival and is associated with particularly grave consequences [3, 40]. A better understanding of the molecular pathways that regulate peritoneal metastasis is essential for the development of novel effective therapies. Growing evidence suggests that lipid metabolism reprogramming plays an important role in tumors grown in organs adjacent to adipose tissues, including colorectal cancer [6, 9, 10], and the TME has also attracted research interest in controlling metastasis by regulating complex interactions between tumor cells and the local milieu. However, whether abnormal lipid metabolism in the TME contributes to peritoneal metastasis of colorectal cancer remains unknown.

A recent report showed that myeloid‐derived suppressor cells (MDSCs) increase fatty acid uptake and FAO to enhance their immunosuppressive functions [41]. Myeloid cells undergo metabolic reprogramming from glycolysis to FAO, a process that is paralleled by the activation of immunosuppressive mechanisms involving arginase I and iNOS and the development of the ability to suppress T‐cell responses [42, 43]. M2 macrophages increase the expression of CD36, which enhances the uptake of VLDL and LDL, activates FAO, and induces characteristic M2 functions [44]. Dendritic cells that accumulated high levels of lipid were found to be ineffective in presenting tumor‐associated antigens, thereby promoting thyroid, kidney, head and neck, and ovarian cancer [45]. The TME can be acidic, hypoxic, and nutrients’ deficiencies, thus causing the metabolism of tumor cells and neighboring stromal cells to be remodeled to facilitate tumor survival, proliferation, and metastasis [46]. All these reports indicated that immune cells or stromal cells in the TME enhance FA catabolism and contribute to tumor progression, metastasis, and poor prognosis.

In our study, the proteomic, RT‐qPCR, IHC, and database analyses highlighted that the expression of CPT1A was low in patients with peritoneal metastatic colorectal cancer and was associated with shorter overall survival. One of the most surprising findings of our present study was that no significant differences in free fatty acids (FFAs) were found between colorectal cancer tissues from patients with PM and those from patients with non‐PM by lipidomics analysis. CAFs, as a major component of the tumor stroma, increase tumor cell motility, metastasis, and invasion through either direct contact or soluble mediators that further activate the surrounding stroma and promote epithelial–mesenchymal transition(EMT) in cancer cells [29, 47, 48]. We predicted that CAFs from patients with PM‐CRC might use FFAs and rely on FAO for their function. Our further experiments showed that overexpression of CPT1A in CAFs could significantly enhance the invasion and metastasis of CRC tumors and increase tumor growth *in vivo*. Consistent with the results, supplementation with OA in the drinking water could increase the growth of subcutaneously inoculated colon carcinoma to a certain extent. Although our data failed to directly demonstrate inhibition of CAF cells in this murine tumor model, it is still possible that FAO inhibition can affect CAF cells that promote tumor growth *in vivo*. Our data suggest that targeting the FA metabolism pathways of CAFs has gained interest because of its potential to uncover novel prevention strategies or therapeutic targets.

It is not clear whether low expression of CPT1A in CRC patients with PM is related to CAFs with CPT1A upregulation, and if so, what regulates the phenomenon. First, we reported that upregulated CPT1A expression in CAFs^PM^ can trigger the metabolic switch from glycolysis to oxidative phosphorylation. Our data implied that the glucose‐dependent metabolic switch from aerobic glycolysis to FAO occurs when less glucose is available in the extracellular tumor milieu. Adiponectin increases fatty acid oxidation by increasing the transcriptional activity of PPARα and the expression of its target genes, including CPT1[49, 50]. We identified one potential mechanism by which CAFs*^CPT1A‐^* ^OE^ can mediate CRC cell‐decreased CPT1A expression through the reduced secretion of adiponectin. Remarkably, upregulation of CPT1A in CAFs promotes the proliferation and invasion of colon cancer by enhancing the ability of CAFs to secrete CCL2, VEGF‐A, and MMP2. Wolf et al identified CCL2 upregulation in metastatic UICC stage IV colon carcinomas [51]. In response to VEGF‐A, the endothelial cells, which are essential to tumor angiogenesis, of adjacent blood vessels migrate and breach the surrounding extracellular matrix, proliferating to form new vascular sprouts that supply the growing tumor [52]. Matrix metalloproteinases (MMPs) are known to play an important role in the metastasis and invasion of tumor cells. MMPs, ECM‐degrading proteases, allow VEGF‐A to interact with VEGF receptors and thus promote angiogenesis [53]. It is likely that the combination of chemokines and cytokines produced by CPT1A^OE^ CAFs plays an important role in CAF‐mediated CRC tumor growth and metastasis. We also demonstrated that tumor cells with CPT1A downregulation induced by CAFs*^CPT1A‐^* ^OE^ increased tumor growth and invasion by increasing glycolysis. Importantly, aerobic glycolysis toward cancerous malignancy is usually associated with tumor recurrence, metastasis, and poor clinical outcome. Overall, CAFs promote metastasis are responsible for creating a premetastatic niche that confers a survival advantage for metastatic cells and thus fosters the organ colonization of metastatic colorectal cancer.

## 5 Conclusions

Our study showed, on the one hand, that the low level of CPT1A in CRC tumors with PM becomes more favorable to increase tumor growth and invasion by increasing glycolysis. On the other hand, energy metabolism switched from glucose to FFA in CAFs^PM^, indicating less glucose utilization and competition. The metabolic balance between CAFs and tumors is not perturbed due to the use of different energy sources, which may be the most effective way to metabolically remodel the tumor microenvironment. Upregulation of CPT1A and increasing FAO in cancer cells confer antiangiogenic drug resistance [9]. However, our data demonstrated that the low level of CPT1A in CRC tumors with PM shifted their metabolism to rely more heavily on glycolysis, which implied that CRC cells with PM may be more sensitive to antiangiogenic drugs and glycolysis inhibitors. Indeed, bevacizumab addition to the first three (CAPOX) or four (FOLFOX/FOLFIRI) neoadjuvant cycles is scheduled to enter clinical trials (NCT02758951) for PM‐CRC patients. Based on our work, it would be interesting to design clinical trials and evaluate the efficiency of adding FAO inhibitors to neoadjuvant chemotherapy for treating human PM‐CRC patients in the future.

Many previous works on metabolism in CAFs have focused on glycolysis. While the role of FAO upregulation in CAFs in colorectal PM had not been investigated until our study, a few limitations of our work do exist. PMs‐CRC are associated with significantly worse prognosis, whether they were the only site of metastasis, or there were also additional sites of metastasis [3]. However, in the present study, the CPT1A expression in patients with peritoneum‐only involvement is significantly lower compared with those with no metastases. It is still unknown whether the low level of CPT1A also happens in PMs‐CRC with additional sites of metastasis, and other tumors with PM, such as ovarian, pancreatic, and gastric cancer. In addition, the mechanism of the oxygen‐independent metabolic switch from aerobic glycolysis to oxidative phosphorylation in CAFs^PM^ is not well defined. Further investigation is required to understand the full molecular mechanism. Our understanding of how competition for resources, such as basic nutrients, is dynamically regulated in a particular niche and how this affects functional changes of cells is just beginning to develop.

## Conflict of interests

The authors declare no conflict of interest.

## Authors contributions

XX Liu conceived and designed the study. XX Liu, SY Peng, Cai J, ZX Yuan, BJ Huang, HM Wang, YC Li, YY Kuang, WF Liang, ZH Liu, and Q Wang contributed to acquisition of data. XX Liu, DC Chen, H Wang, and YM Cui analyzed and interpreted the data. DC Chen and H Wang supervised the study. XX Liu drafted the manuscript.

## Ethics approval and consent to participate

All animal experiments were performed in accordance with a protocol approved by the ethics committee of the Institutional Animal Care of The Sixth Affiliated Hospital, Sun Yat‐sen University, China.

## Consent for publication

Not applicable.

## Supporting information


**Fig. S1.** Glycolysis compensates for FAO loss in CRC cells.
**Fig. S** **2.** Energy was balanced in the primary tumors from patients with peritoneal metastases from colorectal cancer.
**Fig. S3.** CPT1A was upregulated in CAFs from patients with peritoneal metastasis.
**Fig. S4.** CAFs^PM^ promotes CRC cell migration and invasion as detected by Transwell invasion assay
**Fig. S5.** CAFs^PM^ promotes CRC cell migration and invasion as detected by wound healing assay.
**Fig. S6.** Upregulation of CPT1A in CAFs promotes CRC cell migration, invasion and growth.
**Fig. S7.** Upregulation of CPT1A in CAFs promotes CRC cell growth.
**Fig. S8.** CPT1A overexpression in CAFs leads to FAO impairment associated with a metabolic shift towards aerobic glycolysis in colon cancer cells.
**Fig. S9.** Growth of the CRC cells under the indicated conditions was detected by crystal violet staining assay.
**Table S1.** Changes in lipid metabolism pathway protein expression (iTRAQ).
**Table S2.** CPT1A Expression in Colorectal Cancer vs. Normal from Oncomine database.
**Table S3.** The main discriminant metabolites in *CPT1A* ^KD^ DLD1 cells.

## Data Availability

The dataset used and/or analyzed during the current study are available from the corresponding author on reasonable request.
