# Supplementary material for: Enhancing cancer‐associated fibroblast fatty acid catabolism within a metabolically challenging tumor microenvironment drives colon cancer peritoneal metastasis
Source: Mol Oncol. 2021 Feb 16;15(5):1391–411. doi: 10.1002/1878-0261.12917 (PMC8096782; doi:10.1002/1878-0261.12917)
Supplement: Supplementary file 1 — Fig. S1. Glycolysis compensates for FAO loss in CRC cells. Fig. S 2. Energy was balanced in the primary tumors from patients with peritoneal metastases from colorectal cancer. Fig. S3. CPT1A was upregulated in CAFs from patients with peritoneal metastasis. Fig. S4. CAFsPM promotes CRC cell migration and invasion as detected by Transwell invasion assay Fig. S5. CAFsPM promotes CRC cell migration and invasion as detected by wound healing assay. Fig. S6. Upregulation of CPT1A in CAFs promotes CRC cell migration, invasion and growth. Fig. S7. Upregulation of CPT1A in CAFs promotes CRC cell growth. Fig. S8. CPT1A overexpression in CAFs leads to FAO impairment associated with a metabolic shift towards aerobic glycolysis in colon cancer cells. Fig. S9. Growth of the CRC cells under the indicated conditions was detected by crystal violet staining assay. Table S1. Changes in lipid metabolism pathway protein expression (iTRAQ). Table S2. CPT1A Expression in Colorectal Cancer vs. Normal from Oncomine database. Table S3. The main discriminant metabolites in CPT1A KD DLD1 cells. [file MOL2-15-1391-s001.doc]

**Supplementary Figures and Figure legends**

**Figure S1. Glycolysis compensates for FAO loss in CRC cells. (A)** Western blot analysis of CPT1A expression in *CPT1A*^KD^ HCT116 cells. **(B)** Representative fields of colonies of *CPT1A*^KD^ DLD1 cells. **(C)** Representative fields of colonies of *CPT1A*^KD^ HCT116 cells. **(D)** The number of colonies in *CPT1A*^KD^ HCT116 cells(One-way ANOVA). **(E)** The proliferation rate of *CPT1A*^KD^ HCT116 cells evaluated by phase object confluence (%) with the IncuCyte Zoom(One-way ANOVA). Image data for phase object confluence processed by IncuCyte Zoom software. **(F)** Quantitative analysis of total ATP generation in *CPT1A*^KD^ HCT116 cells(One-way ANOVA). **(G)** Pharmacological profile of the extracellular acidification rate (ECAR) monitored with a Seahorse XF24 analyzer for 100 min in *CPT1A*^KD^ HCT116 cells. The metabolic inhibitors glucose, oligomycin A and 2-DG were injected sequentially at different time points as indicated. Bars, mean ± SD. NS indicates no significance, n=3.

**Figure S2. The energy balanced in primary tumors from patients with peritoneal metastases from colorectal cancer.** Expression of the indicated protein signature (the AMP-activated protein kinase, AMPK system) in the primary tumors in patients with PM or non-PM by quantitative proteomics(Student’s t-test).

**Figure S3. CPT1A was upregulated in CAFs from patients with peritoneal metastasis. (A)** Immunofluorescence staining for α-SMA (red) in CAFs which were isolated from a patient with colon cancer. Cells were visualized with a confocal microscope, scale bar is 100 µm. **(B)** The serial sections and immunohistochemical for CPT1A and α-SMA in tumor tissues. The tumor cells (T, blue areas) and CAFs (red areas) were identified, scale bar is 20 µm.

**Figure S4. CAFs^PM^ promotes CRC cell migration and invasion as detected by** **Transwell invasion assay. (A)** Crystal violet staining to quantify DLD1/HCT116 cell Transwell invasion after 24 h of exposure to CAFs^PM^ or CAFs^nPM^. **(B)** The cell number of crystal violet staining to quantify HCT116 cell Transwell invasion after 24 h of exposure to CAFs^PM^ or CAFs^nPM^ and ETO (etomoxir, 50 μM) treatment(One-way ANOVA). **(C)** Crystal violet staining to quantify DLD1/HCT116 cell Transwell invasion after 24 h of exposure to CAFs^PM^ or CAFs^nPM^ under the conditions indicated. **(D)** The cell number of crystal violet staining to quantify HCT116 cell Transwell invasion after 24 h of exposure to CAFs^PM^ or CAFs^nPM^ under the conditions indicated (One-way ANOVA). Bars, mean ± SD. *p < 0.05, **p < 0.01, ***p < 0.001, n = 3.

**Figure S5. CAFs^PM^ promotes CRC cell migration and invasion as detected by** **wound healing assay.** **(A)** Wound healing assay of DLD1 cells incubated with CM (conditioned medium) from the indicated CAFs via IncuCyte ZOOM. **(B)** Wound healing assay of HCT116 cells incubated with CM (conditioned medium) from the indicated CAFs via IncuCyte ZOOM. **(C)** The wound healing ability of HCT116 cells incubated with CM (conditioned medium) from CAFs^PM^ or CAFs^nPM^ and ETO (etomoxir, 50 μM) as detected by IncuCyte ZOOM(One-way ANOVA). Bars, mean ± SD. **p < 0.01, n=3.

**Figure S6. Upregulation of CPT1A in CAFs promotes CRC cell migration, invasion and growth. (A)** The proliferation rate of CAFs*^CPT1A-^*^VC^ or CAFs*^CPT1A-^*^OE^ evaluated by phase object confluence (%) with the IncuCyte Zoom under high-glucose conditions (4.5 g/L) (Student’s t-test). **(B)** The proliferation rate of CAFs*^CPT1A-^*^VC^ or CAFs*^CPT1A-^*^OE^ cells evaluated by phase object confluence (%) with the IncuCyte Zoom under low-glucose conditions (1g/L) (One-way ANOVA). **(C)** Crystal violet staining to quantify DLD1 cell Transwell invasion after 24 h of exposure to CAFs*^CPT1A-^*^VC^ or CAFs*^CPT1A-^*^OE^ under high/low-glucose conditions. **(D)** Crystal violet staining to quantify HCT116 cell Transwell invasion after 24 h of exposure to CAFs*^CPT1A-^*^VC^ or CAFs*^CPT1A-^*^OE^ under high/low-glucose conditions. **(E)** Scatterplots showing the cell number of crystal violet staining to quantify HCT116 cell Transwell invasion after 24 h of exposure to CAFs*^CPT1A-^*^VC^ or CAFs*^CPT1A-^*^OE^ under the indicated conditions. # means compared with the control(Student’s t-test). **(F)** Wound healing assay of DLD1 cells incubated with CM (conditioned medium) from CAFs*^CPT1A-^*^VC^ or CAFs*^CPT1A-^*^OE^ by IncuCyte ZOOM. **(G)** The wound healing ability of HCT116 cells incubated with CM (conditioned medium) from CAFs*^CPT1A-^*^VC^ or CAFs*^CPT1A-^*^OE^ as detected by IncuCyte ZOOM(One-way ANOVA). **(H)** Wound healing assay of HCT116 cells incubated with CM (conditioned medium) from CAFs*^CPT1A-^*^VC^ or CAFs*^CPT1A-^*^OE^ by IncuCyte ZOOM. Bars, mean ± SD. *p < 0.05, **p < 0.01, n=3.

**Figure S7. Upregulation of CPT1A in CAFs promotes CRC cell growth. (A)** The proliferation rate of DLD1 cells evaluated by phase object confluence (%) with the IncuCyte Zoom after incubation with CM (conditioned medium) from CAFs*^CPT1A-^*^VC^ or CAFs*^CPT1A-^*^OE^(One-way ANOVA). **(B)** The proliferation rate of HCT116 cells evaluated by phase object confluence (%) with the IncuCyte Zoom after incubation with CM (conditioned medium) from CAFs*^CPT1A-^*^VC^ or CAFs*^CPT1A-^*^OE^(One-way ANOVA). **(C)** The survival rate of DLD1/HCT116 cells evaluated by CCK-8 assay after incubation with CM (conditioned medium) from CAFs^nPM^ or CAFs^PM^(One-way ANOVA). Bars, mean ± SD. *p < 0.05, **p < 0.01, n=3.

**Figure S8. CPT1A overexpression in CAFs leads to FAO impairment associated with a metabolic shift towards aerobic glycolysis in colon cancer cells.** **(A)** Western blot showing CPT1A expression in DLD1/HCT116 cells cocultured with CAFs*^CPT1A-^*^VC^ or CAFs*^CPT1A-^*^OE^ separated by a Transwell cell culture insert or in CM under low-glucose conditions. **(B)** ECAR and **(C)** OCR was measured under basal conditions and after the addition of the indicated drugs to HCT116 cells by a Seahorse XF24 analyzer. **(D)** ECAR/OCR ratios(One-way ANOVA). ECAR measurement equation used for glycolysis; OCR measurement equation used for basal respiration. **(E)** Wound healing assay of DLD1/HCT116 cells incubated with CM (conditioned medium) from the indicated CAFs and treated with 2-DG (5 mM) via IncuCyte ZOOM. **(F)** The wound healing ability of DLD1 (left panel) and HCT116 (right panel) cells incubated with CM (conditioned medium) from CAFs*^CPT1A^*^-VC^ or CAF*s^CPT1A^*^-OE^ and then treated with 2-DG by IncuCyte ZOOM(One-way ANOVA). n=3. **(H)** Cell viability of HCT116 cells was assessed by annexin-V/PI assay after 48 h treatment with 3-BrPA. The value in each panel indicates the % of survival cells.*p < 0.05, **p < 0.01, ***p < 0.001, n=3.

**Figure S9. Growth of the CRC cells under the indicated conditions was detected by crystal violet staining assay.**

**Supplementary Tables**

**Table S1. Changes in lipid metabolism pathway protein expression (iTRAQ)**

| **Classification** | **Symbol** | **Description** | **Fold change** | **P value** |
| --- | --- | --- | --- | --- |
| Fatty Acid Metabolism | CPT1A CPT2 EHHADH ACOX1 ACOX3 ACACA ACAA2 ACOT13 ACOT11 | carnitine palmitoyltransferase 1A  carnitine palmitoyltransferase 2  enoyl-CoA hydratase and 3-hydroxyacyl CoA dehydrogenase  acyl-CoA oxidase 1  acyl-CoA oxidase 3, pristanoyl  acetyl-CoA carboxylase alpha  acetyl-CoA acyltransferase 2  acyl-CoA thioesterase 13  acyl-CoA thioesterase 11 | -1.546  -2.241  -1.932  -1.836  -3.4289  -1.032  -1.583  -1.748  -17.649 | 0.155  **0.031**  0.101  0.076  0.088  0.963  **0.028**  0.092  **0.034** |
| **Fatty Acid Biosynthesis Regulation** | FASN  PRKAA1  PRKAA2  PRKAB1  PRKACB  PRKAG1  PRKACA | fatty acid synthase  protein kinase AMP-activated catalytic subunit alpha 1  protein kinase AMP-activated catalytic subunit alpha 2  protein kinase AMP-activated non-catalytic subunit beta 1  protein kinase cAMP-activated catalytic subunit beta  protein kinase AMP-activated non-catalytic subunit γ 1  protein kinase cAMP-activated catalytic subunit alpha | -0.960  -1.106  -0.849  -1.941  -1.151  -1.703  -1.238 | 0.912  0.683  0.776  0.330  0.4769  0.279  0.208 |

**Table S2. CPT1A Expression in Colorectal Cancer vs. Normal from Oncomine database**

| **Datasets** | **Cancer type vs. Normal** | **P value** | **Fold change** |
| --- | --- | --- | --- |
| **Kaiser Colon** | Colon Mucinous Adenocarcinomavs vs. Normal | 5.06E-7 | -2.507 |
|  | Rectosigmoid Adenocarcinomal vs. Normal | 3.07E-6 | -2.211 |
|  | Rectal Adenocarcinoma vs. Normal | 6.96E-5 | -2.363 |
| **TCGA** | Colon Mucinous Adenocarcinoma vs. Normal | 2.45E-13 | -2.424 |
|  | Colon Adenocarcinoma vs. Normal | 2.69E-13 | -2.211 |
|  | Rectal Adenocarcinoma vs. Normal | 2.39E-13 | -2.203 |
| **Hong Colorecta** | Colorectal Carcinoma vs. Normal | 1.24E-10 | -2.652 |
| **Skrzypczak** **Colorectal** | Colorectal Carcinomavs. Normal | 5.28E-9 | -2.302 |

**Table S3. The main discriminant metabolites in *CPT1A*^KD^ DLD1 cells**

| **Metabolite name** | **Fold change** | **P-value** |
| --- | --- | --- |
| alpha-ketoglutarate | 0.560018903 | **0.037098443** |
| Oxaloacetate | 0.748257305 | **0.000210529** |
| Succinate | 0.60315954 | **0.004858136** |
| Phosphoenolpyruvate | 1.236781536 | 0.155323344 |
| L-Malic acid | 1.063446715 | 0.523161745 |
| Lactate | 1.683500208 | **2.15253E-06** |
| cis-Aconitate | 1.28344173 | 0.231090788 |
| GMP | 0.331571987 | **6.00472E-07** |
| NAD | 1.085596695 | 0.06571864 |
| Reduced nicotinamide adenine dinucleotide (NADH) | 1.539383779 | **0.000451938** |
| NADP | 1.231093886 | **0.016818708** |
| ADP | 0.935954344 | 0.307429777 |
| Adenosine monophosphate | 0.415252742 | **2.44197E-06** |
| Cyclic AMP | 2.246867937 | **1.06534E-08** |
| Isocitrate | 1.283758112 | 0.170598739 |
| Citrate | 1.146165372 | 0.264547195 |
| Pyruvate | 1.495414793 | **0.010288826** |
| Fumarate | 0.906766769 | **0.014053353** |
| Adenosine 5'-triphosphate (ATP) | 1.688116367 | **0.000477165** |
| Guanosine 5'-diphosphate (GDP) | 0.625516515 | 0.075834419 |
| Guanosine 5'-triphosphate (GTP) | 1.054687285 | 0.789554841 |
| Thiamine pyrophosphate (TPP) | 2.517865957 | **1.03026E-07** |
| Flavin mononucleotide (FMN) | 1.269938632 | **0.041021534** |
| D-Fructose 1,6-bisphosphate | 0.662187586 | 0.051791088 |
| Beta-D-Fructose 6-phosphate | 0.594836325 | **1.88127E-07** |
| 3-Phospho-D-glycerate | 0.974064085 | 0.713428177 |
| D-Glucose 6-phosphate | 4.391807545 | **5.48361E-11** |
| Dihydroxyacetone phosphate | 1.627189915 | **3.15876E-05** |
| Acetyl coenzyme A (Acetyl-CoA) | 0.28245726 | 0.053113809 |
